# Durability and Accelerated Ageing of Natural Fibers in Concrete as a Sustainable Construction Material

**DOI:** 10.3390/ma16216905

**Published:** 2023-10-27

**Authors:** Hafsa Jamshaid, Husnain Ali, Rajesh Kumar Mishra, Shabnam Nazari, Vijay Chandan

**Affiliations:** 1 School of Engineering and Technology, National Textile University, Faisalabad 37610, Pakistan; hafsa@ntu.edu.pk (H.J.); husnaina781@gmail.com (H.A.); 2 Department of Material Science and Manufacturing Technology, Faculty of Engineering, Czech University of Life Sciences Prague, Kamycka 129, 165 00 Prague, Czech Republic; vijay@tf.czu.cz; 3 Department of Sustainable Technologies, Faculty of Tropical AgriSciences, Czech University of Life Sciences Prague, Kamycka 129, 165 00 Prague, Czech Republic; nazari@ftz.czu.cz

**Keywords:** natural cellulosic fibers, fiber-reinforced concrete, basalt, surface degradation, lignocellulose, accelerated aging, fiber pull-out, compressive strength

## Abstract

This paper presents an experimental study on the influence of alkaline environments on natural fibers of plant and mineral origin in concretes. The durability of concrete-based composite materials is influenced by the properties of the reinforcing fiber, and the serviceability of concrete is dependent on its durability. The aim of the present study is to investigate the strength, weight loss %, and surface degradation of jute, sugarcane, coconut, sisal, as well as basalt fibers through an accelerated aging method when used as reinforcements in concrete. The samples were immersed in an alkaline environment of sodium and calcium hydroxide at two different levels of pH for one week. Further, the fibers were immersed in NaOH and Ca(OH)_2_ solutions of 1 M, 2 M, 4 M, and 6 M concentrations for 48 h in order to investigate the gradual effect of an alkaline environment on the mechanical properties of the fiber. It was concluded that the weight loss % was greatest for jute fibers when used in concrete composite, while there was no significant effect on the basalt fiber samples. The strength of jute fiber in the concrete sample was also most severely affected by the aging process, compared to other fibers. The strength of basalt fibers in a concrete composite was least affected by the aging process. In some cases, the sisal fiber sample showed an increase in fiber tenacity after the aging process due to fibrillation, which might have increased the interfacial area. The fiber microstructure before and after the aging was evaluated through the use of scanning electron microscopy (SEM). SEM analyses of different fibers were carried out to investigate surface degradation. The fiber pull-out strength was found to be the greatest for basalt fiber, followed by jute and sisal. This is indicative of the excellent adhesion of such fibers with cement in a concrete composite. In these cases, the use of sisal fiber results in defibrillation and increased specific surface area. Sugarcane and coconut fibers ruptured due to their inherent weakness and provided only a small increment in the mechanical performance of the concrete. Basalt fiber-reinforced concrete offered the greatest compressive strength, followed by jute and sisal. These observations provide crucial information regarding the durability and aging of natural fiber-reinforced concrete.

## 1. Introduction

Concrete is one of the most important construction materials around the globe, but it has some deficiencies, such as brittleness and relatively low impact resistance, tensile strength, fire resistance, durability, and resistance to crack formation. These weaknesses can be overcome by using fibers as reinforcements. In recent years, steel has been used as a reinforcement material in cementitious composite, but it is susceptible to corrosion, which reduces its durability [1]. Many methods are used to overcome this issue, but these may lead to overweight construction structures [2]. In the last decade, scientists have tried to find sustainable, durable, less expensive, and widely available materials for the construction industry. Natural fibers are among the cheapest and most environmentally friendly, light weight, and widely available materials that can be used as reinforcements, providing enough strength to reinforce concrete (TRC) structures. Fibers and textiles can bridge cracks, allowing concrete to withstand deformation. They also improve the ductility of concrete and its load-bearing capacity. Structures made using textile fiber reinforcements are less expensive and more light weight and corrosion resistant. Therefore, they are the perfect means to reduce the amount of steel reinforcement used in cementitious composites [3].

The global construction sector consumes 40% of all materials and energy [3,4]. The primary constituents in a concrete mix are cement, gravel, sand, and steel, which are manufactured at high temperatures, resulting in carbon emissions and high energy consumption. The production of cement is responsible for 7% of the total CO_2_ emissions around the world [5]. The steel used in construction has a high embodied energy and embodied carbon content, i.e., 156,000 MJ/m^3^ and 10,920 kg/m^3^, respectively. In contrast, textile-reinforced concrete (TRC) has a lower embodied energy and carbon value, i.e., 3160 MJ/m^3^ and 340 kg/m^3^, respectively [6]. Researchers have compared textile reinforcements to steel reinforcements, observing that the former provided enough strength to reinforce concrete [7]. Textile materials of different types are added as a partial replacement of cement and aggregates in concrete to improve the properties of the concrete and to reduce environmental pollution. In recent times, the utilization of natural fibers for the reinforcement of concrete has increased. This trend is quite logical, owing to the preference for renewable resources. Its usage reduces the burden of the energy crisis; additionally, it is relatively cheaper, leading to a reduction in foreign reserve expenditure on the import of other fibers, which is especially important for developing countries [8]. Natural fibers are classified according to their origin, i.e., plant-based, mineral-based, and animal-based. Researchers are mostly focusing on plant-based fibers because these are widely available, biodegradable, have lower cost, and show good physical properties. Natural fibers have many advantages, such as their density, price, and properties [9,10,11]. Plant fibers have high potential to be used in cement-based concrete, reducing the carbon footprint and supporting a “go green” approach. Natural fiber can not only enhance the mechanical strength of concrete but can also improve the fatigue life and stiffness of composites. Fibers are extensively used for retrofitting in existing buildings [12,13,14,15]. 

Plant-based natural fibers are classified as stalk/stem/bast fibers (jute, bamboo, etc.), leaf fibers (agave, sisal, etc.) and seed–hair/fruit fibers (cotton, coconut, etc.). Jute is a type of bast fiber that is extracted from the outer cell layer of the plant stem. It can be used as a reinforcement in concrete structures, such as in the case of textile reinforcement concrete (TRC). Many researchers have used jute as a reinforcement in concrete to increase its flexural and tensile strength [16]. Sugarcane is a type of natural fiber which is also known as bagasse. This fiber is made from the stalk/stem of sugarcane plants. These fibers have been used as a reinforcement in TRC samples to improve the physical properties and thermal insulation of concrete structures [17]. Coconut or coir fiber is a type of fruit fiber that is obtained from the outer shell/layer of a coconut. Mature brown fiber is commonly used in mats and sackings. Such fibers can be used as reinforcements in several types of composites, including concrete [18]. Sisal is another type of leaf fiber that has high strength, durability, and resistance to many chemicals. These fibers are used in TRC to enhance the mechanical properties of concrete structures [19,20,21]. Basalt is a mineral-based natural fiber obtained from molten rocks. It has high strength and modulus, is environmentally friendly, and displays higher heat resistance than glass fibers. Basalt fiber is colloquially known as a “21st-century non-polluting green material”. Basalt is used to increase the ductility and flexural strength of concrete [22].

Cement is an alkaline matrix. Ca(OH)_2_ is an inorganic material having a -OH (hydroxyl) group on the surface which migrates to the fiber structure. Cement becomes highly alkaline in wet environments (the pH of wet concrete is between 11 and 13). Several investigations on the long-term performance of concrete reinforced with fibers, especially natural fibers, have been reported [23,24]. However, reinforcing fibers are prone to degradation in the alkaline cementitious environment. Natural fiber-reinforced cementitious composites face durability related challenges. Aging is a critical issue; it leads to a reduction in tensile strength due to pull-out or fracture of the fiber. The durability of different fibers can be predicted accurately in real time, but in practice, it is important to study the accelerated aging of fibers to predict the time-dependent performance of such materials. The durability and performance of textile reinforced concrete under an alkaline environment is characterized by accelerated aging. 

In previous research, accelerated aging of concrete structures reinforced with different materials was reported [25]. The durability and accelerated aging of different fiber reinforced polymer-based reinforcing bars for concrete structures were studied under five different conditions, i.e., water, simulated alkaline solution of two types, saline solution, and combined alkali solution. It was concluded that the strength of the bars reduced after exposure to such environments [26]. Several rebar specimens reinforced with carbon, glass, and aramid fibers in different volume proportions were investigated under various environmental conditions. It was concluded that glass filaments were more prone to reductions in durability than carbon and aramid filaments [27,28,29]. The accelerated aging of sisal fibers under dynamic and static environments such as wetting and drying cycles at different temperatures and humidity conditions was also studied [30]. The durability of sisal and coconut fibers in an alkaline solution of calcium and sodium hydroxide was reported. The aging and durability of concrete reinforced with sisal and coconut fibers under tap water, wetting and drying cycles, and an open-air environment were also studied [31]. Alkaline treatment under aggressive conditions using NaOH and Ca(OH)_2_ resulted in reduced mechanical properties for jute and polyester yarn specimens. Jute yarn underwent hydrolysis and dissolution of lignin, hemicellulose, and cellulose, whereas polyester yarn underwent hydrolysis associated with polymer chain scission [32]. The degradation of concrete reinforced with jute yarns was also studied by researchers. Yarn pull-out tests were performed on concrete structures reinforced with polymer-coated and -uncoated jute yarns. Before the pull-out test, the samples were exposed to accelerated aging, i.e., the concrete samples were cured in water for 28 days; after that, the samples were exposed to a temperature of 40 °C with a relative humidity of 99% for 28, 56, 90, 180, and 360 days. The pull-out test after aging indicated that the coated jute yarn formed stronger bonds compared to the uncoated samples [33].

Based on the available literature, a research gap was identified. The main objective of the present work is to study the ageing of plant and mineral fibers when exposed to an alkaline environment and the effect of this ageing on the durability of concrete materials. The effect of an alkaline environment can be assessed by a comparison of the mechanical performance before and after exposure, as recommended by other researchers [34]. An analysis of the surface morphology was also carried out to investigate the degradation of fibers in an alkaline cementitious environment. Fiber pull-out tests were carried out to determine the surface adhesion and compatibility of the reinforcing fibers with the cement matrix. These studies are essential for the selection of suitable natural fibers of plant and mineral origin for the reinforcement of concrete for more durable and sustainable construction. This work investigates the long-term performance of natural fiber-reinforced concrete as a sustainable construction material.

## 2. Methodology

### 2.1. Materials

**Figure 1:**
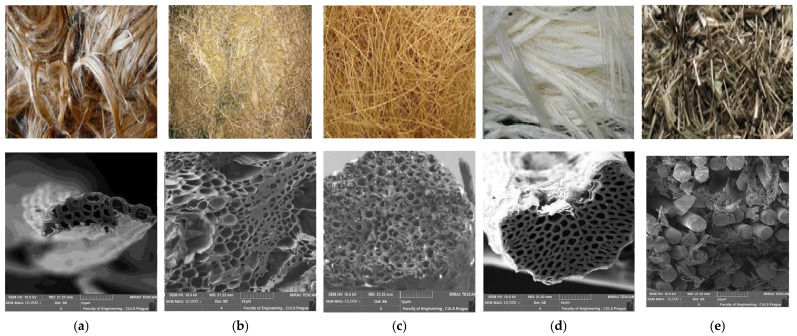
Natural fibers used for concrete reinforcement: (**a**) Jute, (**b**) Sugarcane/bagasse, (**c**) Coconut/coir, (**d**) Sisal, (**e**) Basalt.

Plant-based natural fibers such as jute, sugarcane, coconut, sisal, and mineral fiber, i.e., basalt, were selected for this investigation. The coconut fibers, jute fibers, and sugarcane fibers were purchased from a local market in Faisalabad, Pakistan. Sisal fibers and basalt fibers were supplied by Nanning Jie Cheng Hang Co. Ltd., Nanning, Guangxi, China. Apart from sugarcane fiber (bagasse), none of the materials required further processing. Sugarcane bagasse was obtained from agricultural waste after the juice had been extracted from it. After cleaning and processing, the fibers were cut to the required length of 30 mm [35,36,37]. The fibers used in this work are shown in Figure 1.

Several properties of the fibers used in this research were evaluated in the laboratory, while some others were taken from information provided by the suppliers. Before characterization, all the fibers were conditioned at 25 °C and 65% relative humidity. Fiber properties were measured by ASTM standards [38,39,40,41,42,43,44,45,46,47,48,49,50]. The various properties of the fibers are listed in Table 1.

The alkalis used for the treatment of the fibers were sodium hydroxide and calcium hydroxide (NaOH, Ca(OH)_2_), of analytical grade. These were purchased from a local market in Faisalabad, Pakistan. Although calcium hydroxide is the alkali which is most prevalent in cement, sodium hydroxide was also used to gain a better understanding of the effect of a rigorous alkaline environment on natural fibers. To quantify the limits of the fiber degradation, accelerated aging tests were performed on basalt, jute, sisal, sugarcane, and coconut fibers.

From a chemical point of view, plant fibers consist of cellulose, lignin, and hemicellulose. The rest is pectin, wax, and several other impurities. The characteristics of cellulose, hemicellulose, and lignin in plant fibers play an important role in determining their mechanical, chemical, and thermo-mechanical properties. Cellulose consists of covalent-bonded pyranose rings in each glucose unit. The molecular chains, mutually connected by hydrogen bonds, constitute the microfibers, which are bound to each other by hemicellulose and lignin. Cellulose has a crystalline structure (about 50–90% crystallinity), depending on the source. Due to its structure, cellulose is quite rigid at the molecular level and exhibits relatively higher resistance to tensile stresses. Cellulose is almost insoluble in alkaline solutions, while hemicellulose dissolves easily. Hemicellulose is a branched short-chain polymer which is highly reactive and non-crystalline in nature. Lignin is a hydrophobic natural adhesive that is present in plant fibers and is soluble in alkaline solutions to a limited extent. Lignin actually works as a glue between cellulose and hemicellulose and helps the material to gain rigidity [51,52]. A comprehensive analysis was carried out in order to establish the relationship between ageing effect of fibers and their tensile performance.

Basalt is a mineral fiber with a high silica (SiO_2_) content, followed by Al_2_O_3_, Fe_2_O_3_, Na_2_O, CaO, and MgO. Basalt fibers are composed of several oxides, as shown in Table 2 [53,54,55].

### 2.2. Methods

#### 2.2.1. Accelerated Aging Method

All the fibers were dried (to remove any moisture) and weighed using a digital balance with an accuracy of 0 ± 0.1%. The fiber length was measured carefully. Fiber samples of 30 mm in length were immersed for 1 week in an alkali solution in tight plastic containers to prevent evaporative losses. NaOH solution with 98% concentration and Ca(OH)_2_ with 96% concentration, having pH of 9 and 10, respectively, were used in plastic containers for accelerated aging of the fibers. Further, the fibers were also immersed in NaOH and Ca(OH)_2_ solutions of 1 M, 2 M, 4 M, an 6 M concentrations for 48 h in order to investigate the gradual effect of an alkaline environment on the mechanical properties of the fibers. After removal from the alkali solution, the fibers were neutralized by treatment with acetic acid. Any residual alkaline material was removed by washing the fibers with distilled water. The fibers were dried after treatment in an oven for 30 mins and then further dried in air for 24 h for further investigation. For each sample, 10 measurements were carried out and the average was reported.

#### 2.2.2. Testing

The effect of treatment with NaOH and Ca(OH)_2_ on different fibers such as basalt, jute, sugarcane, sisal, and coconut was studied under different pH levels and different molar concentrations. Notably, the reduction in strength and weight were used to study the effect of alkali treatment on the selected fibers. The strength and weight loss % were measures, and a microscopic analysis of the fiber surfaces before and after chemical exposure was conducted.

#### 2.2.3. Weight Loss %

The test sample were weighed before and after alkali aging to quantify the weight loss. Weight loss of the samples was calculated using the formula:

(1) $$WL\%=\left(\frac{W_{1}-W_{2}}{W_{1}}\right)100\%$$

where *WL* is the weight loss, *W*_1_ is the weight of sample before treatment, and *W*_2_ is the weight of the sample after treatment. For each sample, 10 measurements were carried out and the average was reported.

#### 2.2.4. Mechanical Test of Fibers after Accelerated Aging

The breaking tenacity of different fibers was measured using a universal materials testing machine (Testometric, Rochdale, UK), according to the ASTM D3822M-14 standard [44]. Fiber samples were tested before and after alkali treatment and a comparative analysis was done between control and alkali treated samples. Determining the tensile strength of natural fibers is a problem due to the different cross-section and size of the fibers, their variable dimensions, the influence of local conditions under which a plant is grown (weather, soil, etc.), the procedures used to obtain fibers from a plant, and the maturity degree, etc. The breaking tenacity (cN/tex) was calculated instead of the tensile strength (MPa) to avoid variation due to the above reasons [44]. Tenacity (cN/tex) was used in our comparative analysis. Ten measurements for each fiber were carried out and their averages were reported.

#### 2.2.5. Scanning Electron Microscope for Surface Degradation

SEM was used to investigate the degradation of the surface of fibers after treatment with alkali solutions. SEM images were taken in the range of 100–500× at 100 micrometers. Microstructural images were prepared for different types of fibers before and after the alkali treatments. The samples were prepared with a sputter, i.e., Quorum Q150R ES, which uses gold-plating with an argon gas atmosphere. The thickness of the gold plating was kept at 2 nm using a current of 20 mA. A Quanta 250 scanning electron microscope was used for this purpose. The samples were visualized in a nitrogen atmosphere with a secondary electron (SE) detector, using an acceleration voltage of 20 kV.

#### 2.2.6. Preparation of Cement Matrix and Concrete Blocks

For the concrete samples, ordinary Portland cement (OPC) was used. The conventional procedure of chemical analysis for cements normally involves a tiresome process during the preparation of the samples. In this study, the concentrations of elements in cement were determined by the inductively coupled plasma-optical emission spectroscopy (ICP-OES) method [33,37]. This method is designed to calculate the composition of a broad range of materials, with excellent sensitivity (measurement uncertainty is less than 1%). The results are shown in Table 3. They show good agreement with certified values. The result is related to the pronounced refractory behavior of the elements.

The concrete samples were prepared by mixing the cement and fibers with water, i.e., 60% water, 38% cement and 2% fiber by weight were mixed. The mixture was stirred thoroughly using a glass rod for about 30 min and then allowed to hydrate in sealed plastic containers. Five parts by weight of the concrete mixture (water + cement + fiber) and four parts by weight of alkali were mixed as recommended by the supplier. Then, 2% weight of fiber was added to the concrete mix for each kind of cellulosic and mineral fiber reinforced sample. Further, a reference/control sample was also prepared without fiber. The samples were removed from the mold after 48 h of curing at room temperature and then dried again for 48 h at room temperature.

#### 2.2.7. Fiber Pull-Out Test

**Figure 2:**
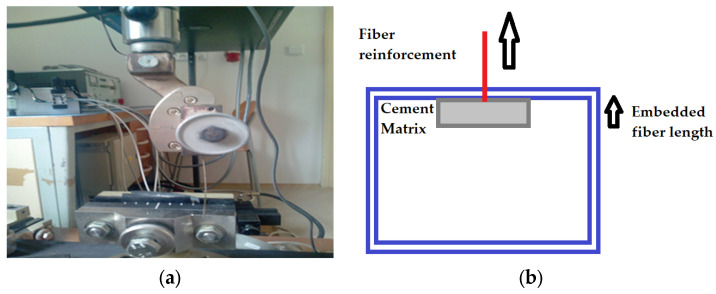
Fiber pull-out test method, (**a**) TIRA tester, (**b**) Experimental set up.

As per the literature, there is no standard method to check the pull-out strength of fiber from concrete. Based on the available related literature, a suitable method was developed to perform these experiments [28,45]. Fibers which were partially immersed/embedded in concrete blocks were pulled using a tensile test set up. This gave a closer approximation of the complex pull-out behavior of the fiber structure present inside a concrete matrix. Sample concrete blocks were prepared with dimensions of 40 × 40 × 10 mm. The fiber was placed in the center of the specimen and was partially embedded. The other end of the fiber was of sufficient size to be clamped and pulled using a TIRA 2300 (LaborTech s.r.o., Opava, Czech Republic) universal testing machine. The fiber was pulled at a speed of 2.0 mm/min. For each type of fiber sample, 10 concrete blocks were prepared and tested. The average value of the pull-out strength was then calculated. The machine used to perform the pull-out tests is shown in Figure 2a, and a schematic of the pull-out procedure shown in Figure 2b.

#### 2.2.8. Compressive Strength of Concrete Blocks

Samples of size 52 mm × 52 mm × 52 mm were prepared for concrete blocks reinforced with 2% weight of natural fibers, as well as plain concrete without any fiber (control sample). The compressive strength was measured using a digital-display hydraulic universal testing machine (Model: Beijing Sinofound WES-100) using test standard ASTM C109/C109M-07 [47]. A loading speed of 1 mm/min was used for the test. Ten samples were tested for each type and the average was calculated.

#### 2.2.9. Data Analysis

Each experiment was executed ten times, and average was considered as the response variable. In order to analyze the effect of alkalinity on fiber tenacity, analysis of variance (ANOVA) was used to ensure the significance of the test results. To evaluate the test data, the Minitab version 21.1.0, Statistical software package was used. The null hypothesis (H0) means a statistically insignificant difference among the measured data with (*p* > 0.05), while the alternate hypothesis (H1) means the opposite of null hypothesis, i.e., a statistically significant difference was observed among the measured data with (*p* < 0.05). The analysis was carried out keeping a 95% confidence interval, which ensures significance of the studied factors if the *p* value is below 0.05.

## 3. Results and Discussion

Degradation of fibers occurs due to exposure to alkaline solutions, which affects the durability of the concrete material. As such, early failure of fibers could reduce the life span of a construction.

### 3.1. Weight Loss (%)

**Figure 3:**
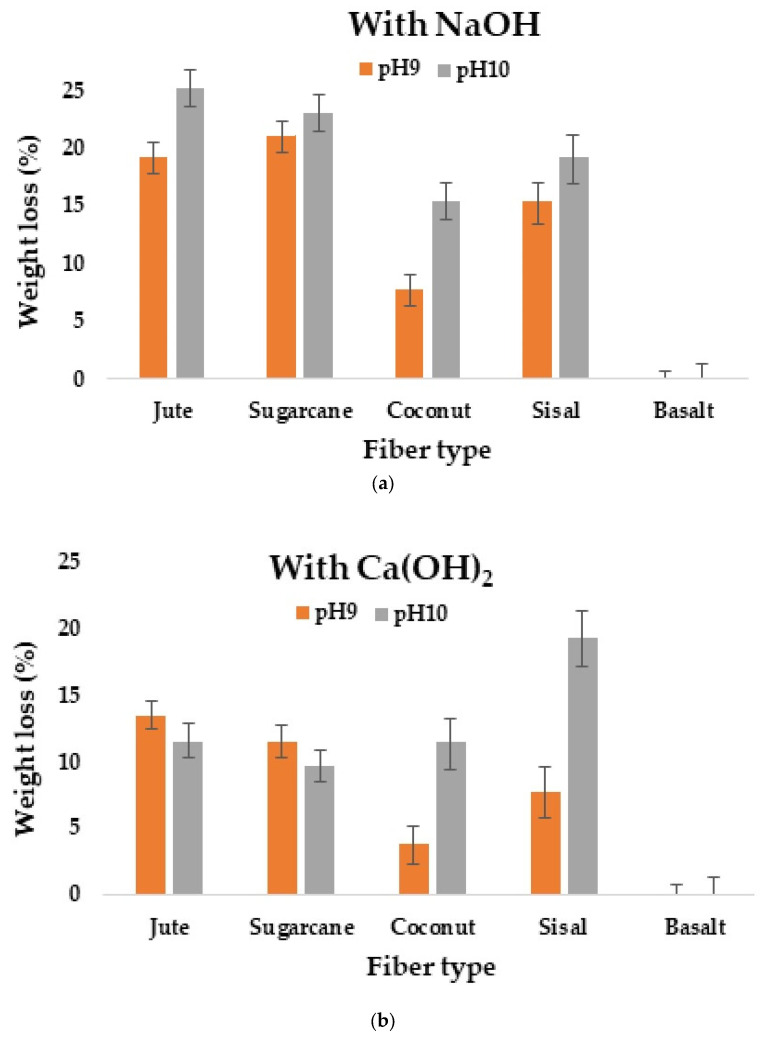
Effect of alkali treatment (**a**) NaOH and (**b**) Ca(OH)_2_ on weight loss of different fibers.

A graphical representation of the weight loss for different types of fibers after alkali treatment is given in Figure 3a,b. The results indicate that the weight loss of the fibers occurred due to exposure to alkali solutions. Higher weight loss occurred at higher pH, especially when exposed to NaOH solution. The weight loss % in jute and sugarcane fibers were higher as compared to those of other plant fibers, but there was no significant weight loss in the case of basalt fiber when exposed to strong alkaline solutions. Weight loss in the cellulosic fibers occurred due to presence of lignin and hemicellulose in the fiber, which are soluble in alkaline solution. When these fibers underwent the accelerated aging process or were exposed to alkali solutions, the hemicellulose and lignin dissolved, which caused a reduction of weight in the fibers. There was no visible/measurable weight loss in the basalt fiber, as lignin and hemicellulose are not present in it.

**Figure 4:**
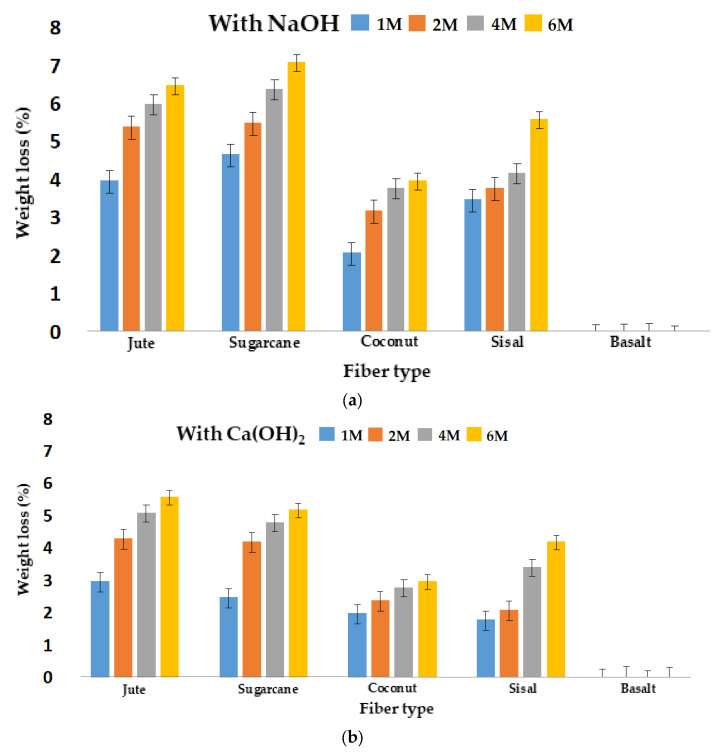
Effect of alkali concentration (**a**) NaOH and (**b**) Ca(OH)_2_ on weight loss of different fibers.

The effect of different molar concentrations of NaOH and Ca(OH)_2_ on the weight loss of different types of fibers is shown in Figure 4a,b, respectively. It shows a gradually increasing weight loss as the concentration of the alkali solution increased. This trend was observed for all the different types of fibers of cellulosic origin. However, the basalt fibers showed no visible weight loss due to their high resistance to alkalis.

It was visible that NaOH at all concentrations had a more severe effect on the natural cellulosic fibers as compared to Ca(OH)_2_.

### 3.2. Mechanical Test of Fibers after Accelerated Aging

**Figure 5:**
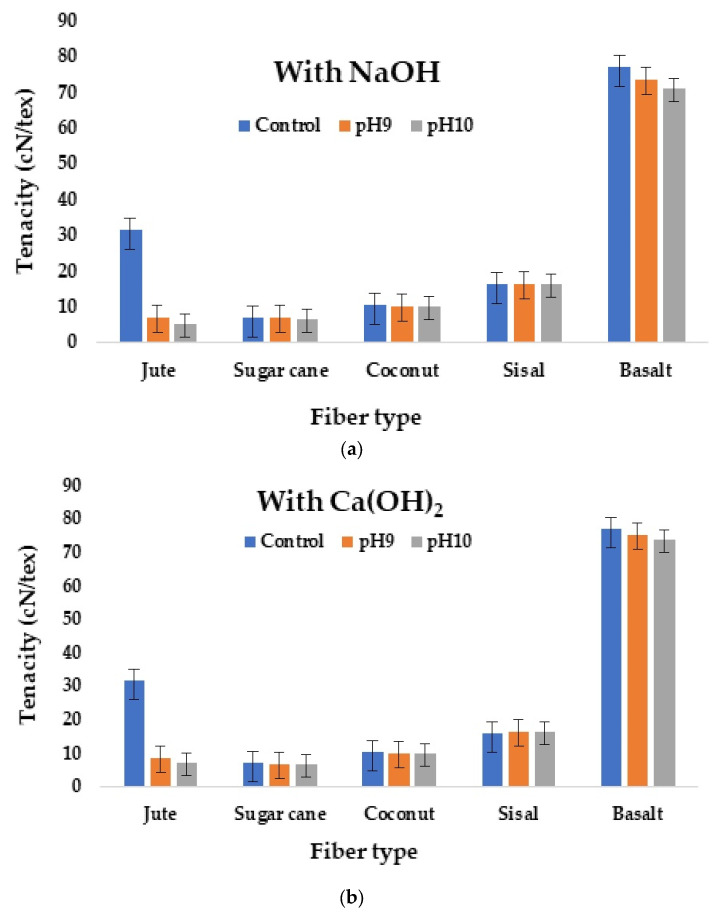
Effect of alkali treatment (**a**) NaOH and (**b**) Ca(OH)_2_ on tenacity of different fibers.

After the accelerated aging process, mechanical tests were conducted to quantify the tenacity of each type of fiber. The tenacity of single untreated fibers was compared with that of alkali treated fibers of the same type. The results demonstrated that the tenacity of the fibers reduced after the aging process. The basalt fibers showed a minimal reduction in mechanical properties as compared to other natural cellulosic fibers. The marginal reduction in mechanical properties in basalt fibers may have been due to their crystallographic structure. The structure of basalt fiber is produced by single tetrahedron olivine, linear chain pyroxenes, and tetrahedral structure gioclase. Some bonds of linear tetrahedrons break down in an alkaline environment, causing a minor reduction in the mechanical properties. The formation of oxides occurs when Fe reacts with O_2_, which might reduce the strength of basalt fibers, even though there was no significant weight loss in basalt fiber, as cations of the reaction mixture bind to the fiber silicate structure. For example, when the fibers were treated with alkali, the network structure broke and cations (Na^+^, Ca^2+^, K^+^, Mg^2+^, Fe^2+^, and Fe^3+^) moved freely in the solution, because Na^+^ and Ca^2+^ cations have comparable dimensions; this is why cations of Na^+^ replace Ca^2+^ in the network structure. These replacements of cations in the network structure of basalt might marginally reduce the mechanical properties but do not affect its weight [56,57,58,59,60]. A graphical representation of the tenacity of different fibers before and after alkali treatment is shown in Figure 5a,b.

**Figure 6:**
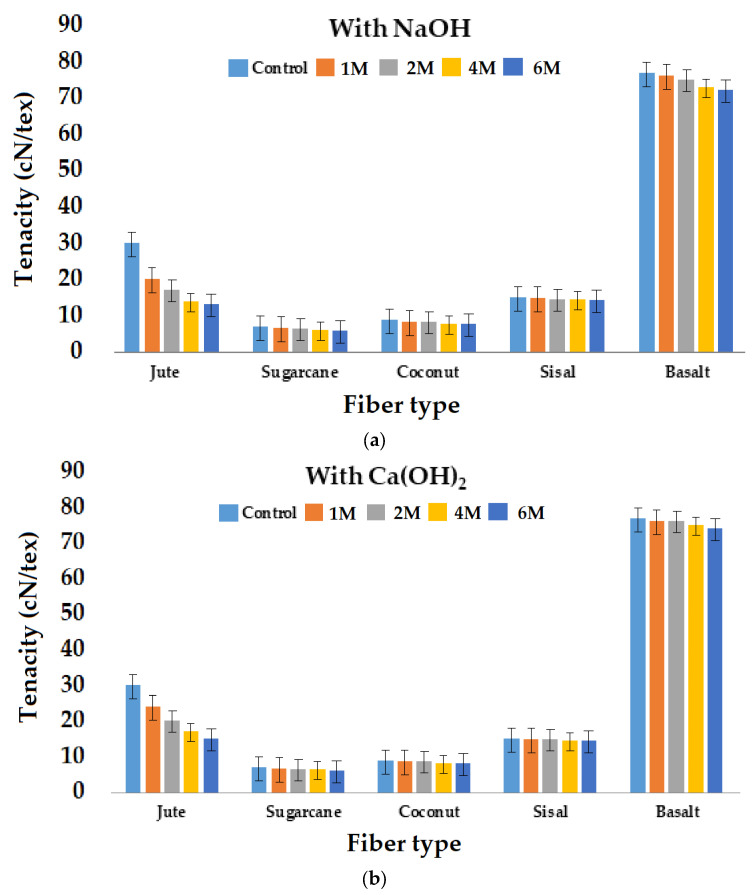
Effect of alkali concentration (**a**) NaOH and (**b**) Ca(OH)_2_ on tenacity of different fibers.

Further, the tenacity of the fibers was also evaluated after treatment with NaOH and Ca(OH)_2_ of 1 M, 2 M, 4 M, and 6 M concentrations for 48 h to investigate the gradual changes. The results are shown in Figure 6a,b.

The strength and stiffness of fibers are directly related to their cellulose content and crystallinity index. A higher crystallinity index means a lower amount of amorphous regions. Fibers exhibit minimum reactivity because they are highly crystalline, thus rendering fewer hydroxyl groups available for reactions with interacting chemicals [61,62,63].

As the cement slurry is a highly alkaline matrix, quick deterioration of mechanical properties of the fibers may occur if they have high hemicellulose content. Mainly, the hemicellulose (the amorphous part of natural fiber) is degraded and lost in a alkaline environment. Free hydroxyl groups of cellulose-OH- result in an undesirable hydrophilic characteristic in fibers. Hydrogen atoms in the free hydroxyl groups are replaced by sodium/calcium atoms. Due to alkali reactions, hydrogen bonds in the crosslinked networks of the cellulose and lignin structure are broken. This makes the fiber soft, which also leads to a decrease in tenacity. In addition, the alkaline condition affects the hydrogen bonding in the chemical structures of natural cellulosic fibers, resulting in an increase in fiber surface roughness. The reaction of cellulose with alkali is as follows:

Cell-OH + NaOH → Cell-O-NA + H_2_O + ⋯ (2)

Among all the plant fibers used, the reduction in mechanical property was maximal in jute, followed by sugarcane. Jute is a lignocellulosic fiber in which the lignin content is 13.1%, the cellulose content 62%, and the hemicellulose content is 24%. The alkaline solution dissolved the hemicellulose present in the jute fibers. Strong alkaline solutions also cause the hydrolysis of cellulose, which results in the degradation of the fibers [62,63,64,65]. After NaOH treatment, the hemicellulose and a part of the lignin were removed from the cell walls of the fibers. The alkali treatment broke or removed the semicrystalline structure of jute fibers, which resulted in a decrease in mechanical properties. The decrease in the mechanical properties was most pronounced in the jute fiber, as it is the cellulosic fiber with the highest hemicellulose content. Sugarcane bagasse fiber also has a relatively high content of hemicellulose, but also has a high content of lignin, which provides rigidity to the fiber. Coir fiber has a relatively low amount of hemicellulose and high amount of lignin, which is poorly soluble in alkaline solutions. Therefore, it is stronger but less flexible [66]. In several cases, sisal fibers showed a slight increase in mechanical properties as the fibers underwent the fibrillation process. Fibrillation is a process in which fibers develop a hairy appearance [67,68]. 

It was evident that NaOH caused more severe damage to the cellulosic fibers and thus caused reduction in tenacity compared to treatment with Ca(OH)_2._ These observations were consistent with previous results [68,69,70]. Basalt fibers showed excellent alkali resistance, maintaining almost all of their tenacity. Such behavior was observed after treatment with NaOH and Ca(OH)_2_ in 1 M, 2 M, 4 M, and 6 M concentrations for 48 h and after 1 week treatment with both alkalis at high concentrations. These results indicate the excellent performance of mineral fibers, e.g., basalt, as reinforcement materials in concrete.

The ANOVA results showed that the alkali concentration and its pH had significant effects on the mechanical properties of fibers used in concrete. The *p* value of the results was α = 0.00, which is below 0.05, and clearly shows that the results are significant and the effect of accelerated aging on fibers is significant (R square is 95.41%). A greater R-square indicates greater effectiveness of the treatment.

### 3.3. Scanning Electronic Microscopy (SEM)

**Figure 7:**
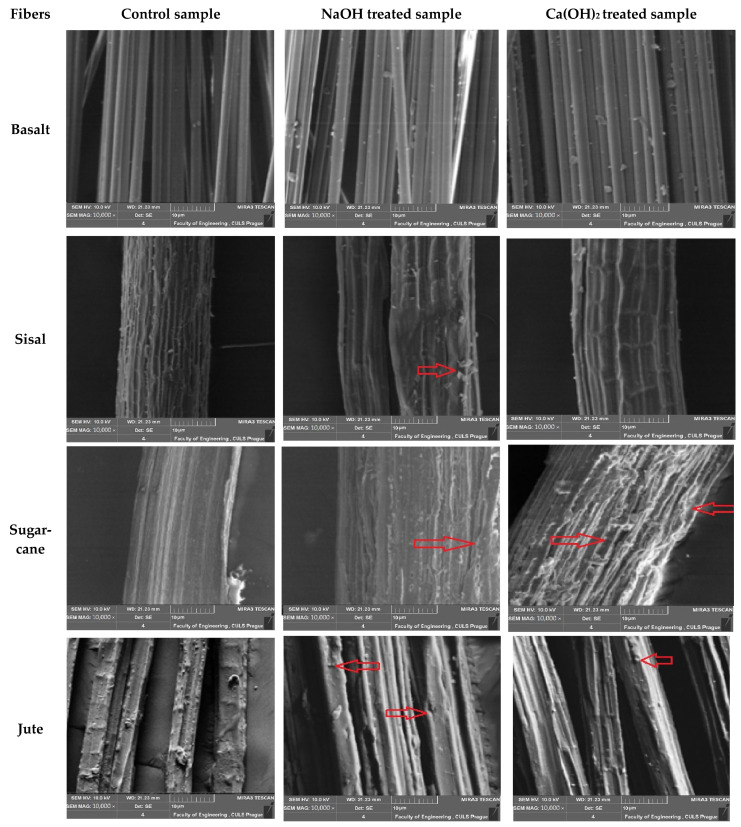

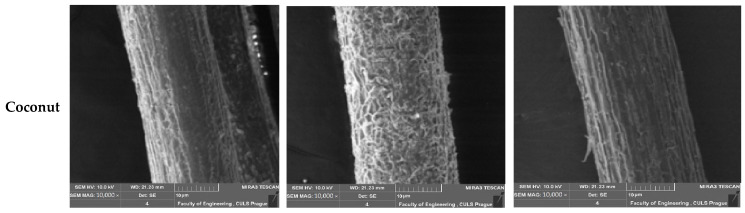
SEM images of natural fibers with and without accelerated aging. Red arrows show the micro-cracks.

The surface morphology of the fibers was also investigated using scanning electronic microscopy. Microscopic images of different types of fibers before and after alkali treatment are shown in Figure 7. The microscopic images showed that the effect of alkali treatment was more apparent on jute fiber as compared to other types of fibers. The jute fibers after exposure to an alkaline environment underwent a significant reduction in diameter and severe degradation of the surface. Small holes were visible in the surface of the jute fibers. The SEM images showed that the lumens of jute fibers collapsed after the aging process, reducing their permeability. The lumens or side walls of the fibers are responsible for the capillary pressure or permeability of the fibers. The untreated jute fiber showed open lumens, as compared to aged/alkali-treated jute fibers. The SEM images showed micro-cracks on the surface of fibers, which were responsible for a reduction in the mechanical properties of those fibers.

### 3.4. Fiber Pull-Out from Concrete

The bonding between the concrete matrix and the reinforcing fibers determines the pull-out behavior. A strong adhesion and interfacial bonding between the reinforcing fibers and the concrete or cement matrix is key to stability and performance under tensile/compressive load. Weaker bonding may lead to deboning and easy pull-out of the fibers. If the interfacial strength between the fibers and the matrix is too high, the fibers might rupture when there is crack initiation. In the opposite case, if the interface is too weak, the fiber reinforcement is easily pulled out of the concrete. Therefore, the bond strength determines the mechanical properties of fiber-reinforced concrete. Pull out tests are useful to obtain information about the load transfer between the cement matrix and the fiber reinforcement. These tests characterize both pull-out and rupture of the textile fiber as different failure modes. The peak load is not necessarily the breaking point of the fiber itself, but rather, the load at initiation of cracking [71]. Traditionally, the quality of interfacial bonding is calculated by the apparent interfacial shear strength (apparent IFSS, *f_app_*), [72].

*ґ_app_* = *F_max_*/(*πdl_e_*) (3)

where *d* designates the fiber diameter, and *l_e_* is the embedded fiber length inside concrete block.

The *r_app_* values calculated using Equation (3) usually distinguish between “good” and “poor” bond strengths and provide an estimation of the efficiency of interfacial bonding between the cement matrix and the fiber. The *r_app_* values obtained for the different types of fibers are given in Table 4; the values presented here are the averages of 10 measurements.

In the case of basalt fibers, very small slippage (displacement) was observed, as they have excellent adhesion with the cement matrix. The maximum tensile stress was the highest in the case of basalt fibers. A similar situation was observed in the case of jute fibers, but in this case, the maximum force recorded was much lower than that of basalt, as jute fiber has an overall lower strength value. In case of sisal and sugarcane, much higher slippage/displacement/deformation was observed. Such large displacements prior to material failure are crucial with regard to structural safety as well as energy dissipation, particularly in the case of dynamic loading in concrete. Higher strength (***ґ_app_***) levels can be reached at high levels of deformations. It was observed that the adhesion performance of basalt and jute fibers was superior to that of other types of fibers. This could be predicted based on the basic of mechanical properties of such fibers and the Halpin-Tsai equations governing the performance of composites/concrete reinforced with fibers. The Halpin-Tsai model is commonly used to predict the compressional strength and modulus for composites with defined fiber alignments [73,74]. The Halpin-Tsai equations are defined as:

(4) $$K_{c}=K_{m}\left[\frac{1+\xi \zeta V_{f}}{1-\eta V_{f}}\right]$$

(5) $$\mathrm{With}\;\eta =\left[\frac{\left(K_{f}/K_{m}\right)-1}{\left(K_{f}/K_{m}\right)+\zeta }\right]$$

where *K_c_* is the effective compressional property of the composite/concrete, while *K_f_* and *K_m_* are the corresponding fiber and matrix compressional properties; *V_f_* denotes the fiber volume fraction, and *ζ* is a geometrical parameter which represents the reinforcement geometry, packing geometry, and the loading conditions.

The surface morphology of jute fibers is also responsible for its strong interface with concrete. Sisal fibers also present a relatively high pull-out strength, which is a result of fibrillation and an increase in the interfacial surface area. The experimental findings are given in Table 5.

**Figure 8:**
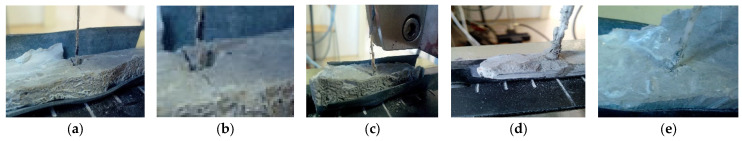
Photographic images of fiber pull-out/rupture (**a**) Basalt, (**b**) Jute, (**c**) Sisal, (**d**) Sugarcane/bagasse, (**e**) Coconut/coir.

The photographs in Figure 8 show that overall, there was adequate adhesion between the fibers and the concrete block. The force was transmitted to the reinforcing fiber by friction with the concrete. The load was transferred onto the fibers from the cement matrix based on adhesion/friction, depending on the nature of the bond. The nature of the bonding depends on the fiber surface morphology and varies along the length of natural cellulosic fibers. This leads to a complex failure mechanism involving partial rupture and pull-out of the fibers. The pull-out was observed as a microscopic failure (i.e., partial fiber rupture and partial pull-out).

Degradation of the cellulosic fibers was observed due to the alkalinity in the cement. This enhanced the durability of the concrete in the construction only marginally. Calcium hydroxide is the primary alkali in cement. We observed higher weight loss with longer treatment time, higher pH, and even by using a relatively stronger alkali, i.e., NaOH. There was negligible weight loss in the case of basalt fiber, since it was not affected by the alkali treatment. The pull-out strength results for the untreated fiber samples were compared with those of the alkali treated samples. The pull-out strength was found to be higher in terms of maximum load and minimum elongation %. Jute fiber also proved to be the second best among the selected fibers with respect to alkali resistance. This was also observed in the SEM images (Figure 7). The microstructure indicated maximum degradation in the cases of sugarcane and coconut fibers. In those cases, the adhesion of fibers with the cement was also good, but the weakness of the individual fibers led to ruptures. This can be attributed to the high slippage/displacement/deformation that occurred during pull-out.

### 3.5. Compressive Strength

The compressive strength of concrete blocks reinforced with 2% weight of the fibers was measured. The results for the basalt, jute, sisal, sugarcane, and coconut fiber reinforced concrete samples were compared with those of the control sample (without fiber). The results are given in Table 6.

**Figure 9:**
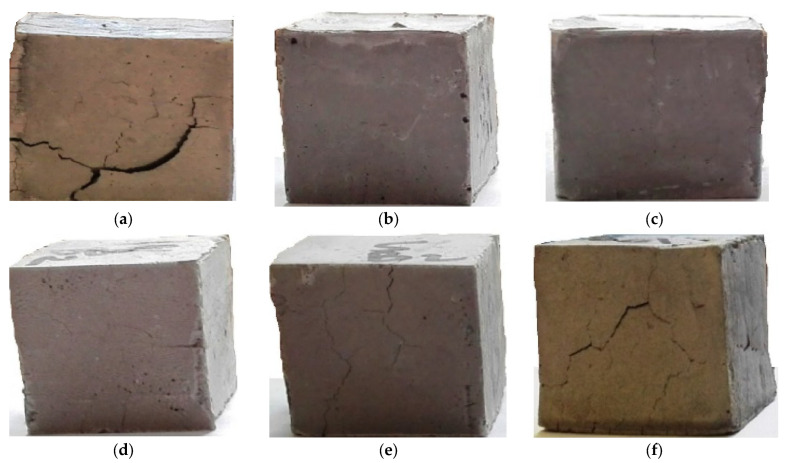
Sample concrete blocks after compression and cracking, (**a**) Control, (**b**) Basalt, (**c**) Jute, (**d**) Sisal, (**e**) Coconut/coir, (**f**) Sugarcane/bagasse fiber reinforced concrete blocks.

For all the concrete samples reinforced with 2% weight of different types of fibers (coconut, sugarcane bagasse, jute, and sisal), the compressive strength was higher than in the control sample. The maximum compressive strength was observed for the 2% basalt fiber-reinforced concrete sample, followed by the jute and sisal fiber-based samples. The blocks prepared with sugarcane and coconut fiber-based concrete exhibited the lowest compressive strengths, even though these values were higher than those of the control sample. These findings are in accordance with theoretical/numerical estimations based on the Halpsin-Tsai models [30,32,45,70,71,72,73,74]. The fiber mechanical properties and their durability significantly influenced the compressive strength of the concrete. This held true for all types of cellulosic and mineral fibers. The stability of basalt was again ascertained by the minimal crack length observed in jute, sisal, coconut, and sugarcane fiber-based concrete composites. The fibrillation of sisal might have helped to restrict crack propagation. Sugarcane and coconut fibers could not prevent cracking very significantly due to fiber degradation in the alkaline environment of cement. However, their performance was visibly superior to that of the control sample. The sample concrete blocks reinforced with 2% weight of different types of cellulosic and mineral fibers showed in an improvement in compressive performance, are shown in Figure 9.

The inherent mechanical properties of the fibers seemed to improve the compression behavior of concretes reinforced with different types of cellulosic/mineral fibers. These observations were consistent with the results of the Halpin-Tsai equations and findings from other researchers [30,32,45]. Basalt, jute, and sisal fibers had superior mechanical properties, as indicated in Table 1. The coconut and sugarcane fibers were found to be relatively weaker and thus could not provide enough strength enhancement to concrete. This trend was consistent with the pull-out performance observed for all types of fibers with 2% weight fraction [52]. With higher fiber loadings, there may be problems associated with the flow behavior of concrete [55,56,57,58,59,60].

## 4. Conclusions

Fiber reinforced concrete is of prime importance in the construction sector. Fiber is used as a reinforcement in concrete retrofitting, serving as an internal and external reinforcement in concrete structures. It is gradually becoming an important part of the construction industry. Different lignocellulosic fiber materials are found all over the world, either as wood or agricultural waste. In this study, the durability of different lignocellulosic fibers and mineral fibers were investigated, and tests were performed to quantify the weight and strength loss of the fibers before and after undergoing an accelerated aging process. Based on the results, the following conclusions are drawn. The percentage of weight lost after the aging process was greatest in the cases of jute and sugarcane fibers. There was no significant weight loss in the case of basalt fibers after the aging process. The tenacity of jute fibers was affected the most after the aging process. Coconut/coir fibers showed minimum reductions in strength, while the tenacity of sisal fibers sometimes increased after the aging process due to fibrillation. SEM images showed degradation of the fiber surface, a reduction in diameter, and the development of small voids on the surface of fibers after the aging process, especially in the case of jute fibers. It is important to consider the chemistry of plant fibers to achieve greater durability of concrete. The pull-out strength was found to be greatest for basalt, followed by jute and sisal fibers. This is indicative of the excellent adhesion of such fibers with the cement in a concrete composite. Sisal fibers resulted in defibrillation and an increase in specific surface area, thus increasing the interfacial strength. Sugarcane and coconut fibers ruptured due to their inherent weaknesses. The compressive strength of basalt fiber-based concrete was found to be the greatest, followed by jute and sisal based concrete samples. The experimental findings were validated by numerical/theoretical models based on Halpin-Tsai equations. These observations broaden our understanding of the compatibility of natural-origin cellulosic as well as mineral fibers in concrete materials and help in determining the durability and aging of natural fiber-reinforced concrete under alkaline conditions. Natural fiber reinforced concrete may be an alternative sustainable construction material in the future.

## Figures and Tables

**Table 1:** Properties of fibers.

Fiber Characteristics	Jute	Sisal	Sugarcane	Coconut	Basalt
Fiber Diameter (µ/micron)	17.4 ± 1.4	20 ± 1.2	22 ± 1.2	21 ± 1.1	10 ± 1.1
Fiber Fineness (Tex, g/km)	17.2 ± 1.2	21.1 ± 1.1	32 ± 1.2	31	12 ± 1.3
Fiber Length (mm)	32 ± 2.1	30.2 ± 2.1	30 ± 2	30 ± 2	30 ± 2
Aspect ratio (length/dia.)	164–345	210–410	136–318	144–428	254–265
Fiber Density (g/cm^3^)	1.32	1.5	0.82	1.3	2.63
Volume Porosity (%)	16–18	11–15	39–42	31–34	NA
Cellulose content (%)	61–72	67-79	45–55	31–45	NA
Lignin content (%)	12–13	8–11	19–24	42–46	NA
Crystallinity (%)	60–65	68–70	51–53	26–34	85–95
Angle of orientation (°)	8–10	10–25	14–15	31–48	65–75
Tensile strength (MPa)	480 ± 16.2	380.4 ± 22.4	68 ± 9.1	176 ± 7.8	1542 ± 19.1
Elongation at break (%)	2.3 ± 0.1	2.35 ± 0.2	1.5 ± 0.1	3.7 ± 0.3	1.3 ± 0.1
Fiber Modulus (GPa)	37.5 ± 1.4	27.5 ± 0.4	18.7 ± 0.8	21.7 ± 0.3	562 ± 12.1
Fiber Tenacity (cN/Tex)	30.2 ± 1.5	15.1 ± 0.7	7.2 ± 0.4	9.2 ± 0.7	77 ± 2.7

**Table 2:** Chemical composition (%) of basalt fiber.

SiO_2_	Al_2_O_3_	CaO	MgO	K_2_O	Na_2_O	Fe_2_O_3_	TiO_2_
48.39	16.7	7.7	4.7	1.6	3	15.3	3.8

**Table 3:** Metal components and their content (concentration) in the used cement.

Components	In (mg/kg)
Be	1.11
Cu	172
Cr	75.4
Ni	45.4
Pb	73.2
Fe	16,777
Mg	10,178
Ca	415,569
Na	2042
Mn	525
Al	23,497
B	170,867
Ti	1586
V	39.5
Zn	274
Sr	773
Ba	341
K	7882

**Table 4:** Apparent interfacial shear strength (*r_app_*) for different fibers in cement.

Reinforcement Fiber Type	App *ґ_app_* (MPa)
Basalt	1.769 ± 0.11
Jute	0.991 ± 0.05
Sisal	0.872 ± 0.05
Sugarcane	0.663 ± 0.07
Coconut	0.745 ± 0.08

**Table 5:** Experimental findings of the fiber pull-out test.

Reinforcement Fiber Type	Peak Force *F_max_* (N)	Crack Opening Displacement at Peak Force (mm)	Failure Mechanism
Basalt	82.90 ± 3.11	2.56 ± 0.11	Micro-cracking in concrete
Jute	53.54 ± 1.12	21.19 ± 1.05	Fiber pulled out
Sisal	52.61 ± 1.11	10.84 ± 0.78	Fiber pulled out
Sugarcane	25.68 ± 1.01	12.30 ± 0.88	Fiber ruptured
Coconut	27.51 ± 1.00	14.20 ± 0.95	Fiber ruptured

**Table 6:** Compressive strength for different concrete samples.

Reinforcement Fiber Type	Compressive Strength (MPa)
None (control)	3.26 ± 0.22
Basalt	10.47 ± 1.53
Jute	6.75 ± 0.74
Sisal	5.52 ± 0.52
Sugarcane	4.05 ± 0.87
Coconut	4.34 ± 0.43

## Data Availability

Data sharing not available.

## References

[1] Hasan K.M.F., Horváth P.G., Alpár T. (2022). Lignocellulosic fiber cement compatibility: A state of the art review. J. Nat. Fibers.

[2] Torgal F.P., Jalali S. (2011). Natural Fiber Reinforced Concrete, No. 1994.

[3] Silveira D., Varum H., Costa A., Martins T., Pereira H., Almeida J. (2012). Mechanical properties of adobe bricks in ancient constructions. Constr. Build. Mater..

[4] Torgal F.P., Jalali S. (2012). Earth construction: Lessons from the past for future eco-efficient construction. Constr. Build. Mater..

[5] Zakaria M., Ahmed M., Hoque M., Islam S. (2016). Scope of using jute fiber for the reinforcement of concrete material. Text. Cloth. Sustain..

[6] Bourmaud A., Baley C. (2012). Nanoindentation contribution to mechanical characterization of vegetal fibers. Compos. Part B Eng..

[7] Chandramohan D., Marimuthu K. (2011). A Review on Natural Fibers. Sci. Res..

[8] Pérez E., Famá L., Pardo S.G., Abad M.J., Bernal C. (2012). Tensile and fracture behaviour of PP/wood flour composites. Compos. Part B Eng..

[9] Shih Y.F., Cai J.X., Kuan C.S., Hsieh C.F. (2012). Plant fibers and wasted fiber/epoxy green composites. Compos. Part B Eng..

[10] Jamshaid H., Mishra R.K., Raza A., Hussain U., Rahman M.L., Nazari S., Chandan V., Muller M., Choteborsky R. (2022). Natural Cellulosic Fiber Reinforced Concrete: Influence of Fiber Type and Loading Percentage on Mechanical and Water Absorption Performance. Materials.

[11] Milanese A.C., Cioffi M.O.H., Voorwald H.J.C. (2012). Thermal and mechanical behaviour of sisal/phenolic composites. Compos. Part B Eng..

[12] Huang J., Rodrigue D. (2022). Stiffness Behavior of Sisal Fiber Reinforced Foam Concrete under Flexural Loading. J. Nat. Fiber..

[13] Huang J., Tian G., Huang P., Chen Z. (2020). Flexural Performance of Sisal Fiber Reinforced Foamed Concrete under Static and Fatigue Loading. Materials.

[14] Khan M., Ali M. (2019). Improvement in concrete behavior with fly ash, silica-fume and coconut fibres. Constr. Build. Mater..

[15] Frappa G., Pauletta M. Seismic retrofitting of a reinforced concrete building with strongly different stiffness in the main directions. Proceedings of the 14th Fib International PhD Symposium in Civil Engineering.

[16] Messiry M.E., Fadel N. (2021). Tailoring the mechanical properties of jute woven/cement composite for innovation in the architectural constructions. J. Nat. Fibers.

[17] Palanikumar K., Ramesh M., Reddy K.H. (2016). Experimental Investigation on the mechanical properties of green hybrid sisal and glass fiber reinforced polymer composites. J. Nat. Fibers.

[18] Kavitha S., Kala T.F. (2018). A review on natural fibers in the concrete. Int. J. Adv. Eng. Technol..

[19] Elshazli M.T., Ramirez K., Ibrahim A., Badran M. (2022). Mechanical, Durability and Corrosion Properties of Basalt Fiber Concrete. Fibers.

[20] Palanisamy E., Ramasamy M. (2022). Dependency of sisal and banana fiber on mechanical and durability properties of polypropylene hybrid fiber reinforced concrete. J. Nat. Fibers.

[21] Rajendran M., Nagarajan C. (2022). Experimental investigation on bio-composite using jute and banana fiber as a potential substitute of solid wood-based materials. J. Nat. Fibers.

[22] Jaballi S., Miraoui I., Hassis H. (2017). Long-unidirectional palm and sisal fibers reinforced composite: An experimental investigation. J. Nat. Fibers.

[23] Prasanthi P.P., Babu K.S., Kumar M.S., Kumar A.E. (2021). Analysis of sisal fiber waviness effect on the elastic properties of natural composites using analytical and experimental methods. J. Nat. Fibers.

[24] Zakaria M., Ahmed M., Hoque M., Shaid A. (2020). A Comparative study of the mechanical properties of jute fiber and yarn reinforced concrete composites. J. Nat. Fibers.

[25] Ahmad S., Khushnood R.A., Jagdale P., Tulliani J.M., Ferro G.A. (2015). High performance self-consolidating cementitious composites by using micro carbonized bamboo particles. Mater. Des..

[26] Rashid K., Balouch N. (2017). Influence of steel fibers extracted from waste tires on shear behavior of reinforced concrete beams. Struct. Concr..

[27] Rashid K., Nazir S. (2018). A sustainable approach to optimum utilization of used foundry sand in concrete. Sci. Eng. Compos. Mater..

[28] Ayub T., Shafiq N., Nuruddin M.F. (2014). Mechanical properties of high-performance concrete reinforced with basalt fibers. Procedia Eng..

[29] Ali M. (2014). Seismic performance of coconut-fiber-reinforced-concrete columns with different reinforcement configurations of coconut-fiber ropes. Constr. Build. Mater..

[30] Elsaid A., Dawood M., Seracino R., Bobko C. (2011). Mechanical properties of kenaf fiber reinforced concrete. Constr. Build. Mater..

[31] Beskopylny A.N., Stel’makh S.A., Shcherban E.M., Mailyan L.R., Meskhi B., Shilov A.A., Beskopylny N., Chernil’nik A. (2022). Enhanced Performance of Concrete Dispersedly Reinforced with Sisal Fibers. Appl. Sci..

[32] Thanushan K., Yogananth Y., Sangeeth P., Coonghe J.G., Sathiparan N. (2021). Strength and durability characteristics of coconut fibre reinforced earth cement blocks. J. Nat. Fibers.

[33] Jamshaid H., Mishra R., Noman M.T. (2018). Interfacial performance and durability of textile reinforced concrete. J. Text. Inst..

[34] Alengaram U.J., Al Muhit B.A., Bin Jumaat M.Z. (2013). Utilization of oil palm kernel shell as lightweight aggregate in concrete—A review. Constr. Build. Mater..

[35] Muda Z.C., Syamsir A., Mustapha K.N. (2016). Impact resistance behaviour of banana fiber reinforced slabs. IOP Conf. Ser. Earth Environ. Sci..

[36] Pajak M., Ponikiewski T. (2017). Experimental investigation on hybrid steel fibers reinforced self-compacting concrete under flexure. Procedia Eng..

[37] Jamshaid H., Mishra R., Militky J., Pechociakova M., Noman M.T. (2016). Mechanical, thermal and interfacial properties of green composites from basalt and hybrid woven fabrics. Fiber. Polym..

[38] (2018). Standard Specification for Fineness of Types of Alpaca.

[39] (2021). Standard Test Method for Assessing Clean Flax Fiber Fineness.

[40] (2018). Standard Test Method for Length and Length Distribution of Manufactured Staple Fibers (Single-Fiber Test).

[41] (2016). Standard Specification for Steel Fibers for Fiber-Reinforced Concrete.

[42] (2018). Standard Test Methods for Density Determination of Flax Fiber.

[43] (2016). Standard Test Methods for Constituent Content of Composite Materials.

[44] (2020). Standard Test Method for Tensile Properties of Single Textile Fibers.

[45] (2017). Standard Test Method for Splitting Tensile Strength of Cylindrical Concrete Specimens.

[46] (2021). Standard Test Method for Flexural Strength of Concrete (Using Simple Beam with Third-Point Loading).

[47] (2021). Standard Test Method for Compressive Strength of Hydraulic Cement Mortars (Using 2-in. or [50-mm] Cube Specimens).

[48] (2013). Standard Test Method for Determining Potential Resistance to Degradation of Pervious Concrete by Impact and Abrasion.

[49] (2017). Standard Test Method for Density, Relative Density (Specific Gravity), and Absorption of Coarse Aggregate.

[50] (2020). Standard Tables of Commercial Moisture Regains and Commercial Allowances for Textile Fibers.

[51] Bala S., Chandrashekaran J., Selvan S.S. (2015). Experimental investigation of natural fiber reinforced concrete in construction industry. Int. Res. J. Eng. Technol..

[52] Choi S.Y., Park J.S., Jung W.T. (2011). A study on the shrinkage control of fiber reinforced concrete pavement. Procedia Eng..

[53] Stephens D. Natural fiber reinforced concrete blocks. Proceedings of the 20th WEDC Conf Affordable Water Supply and Sanitation.

[54] Nazmul R.T., Sainsbury B.-A., Al-Deen S., Garcez E.O., Ashraf M. (2023). An Experimental Evaluation of Hemp as an Internal Curing Agent in Concrete Materials. Materials.

[55] Feng J., Sun W., Zhai H., Wang L., Dong H., Wu Q. (2018). Experimental study on hybrid effect evaluation of fiber reinforced concrete subjected to drop weight impacts. Materials.

[56] Marar K., Eren O., Çelik T. (2001). Relationship between impact energy and compression toughness energy of high-strength fiber-reinforced concrete. Mater. Lett..

[57] Ramakrishna G., Sundararajan T. (2005). Impact strength of a few natural fiber reinforced cement mortar slabs: A comparative study. Cem. Concr. Compos..

[58] Rehacek S., Simunek I., Kolisko J., Hunka P. Impact resistance of steel fiber reinforced concrete. Proceedings of the Fibre Concrete 2011.

[59] Mishra R., Petru M., Rojas I., Castillo-Secilla D., Herrera L.J., Pomares H. (2021). Natural Cellulosic Fiber Reinforced Bio-Epoxy Based Composites and Their Mechanical Properties. Bioengineering and Biomedical Signal and Image Processing.

[60] Ghulam M.A., Uddin M., Jamshaid H., Raza A., Tahir Z.R., Hussain U., Satti A.N., Hayat N., Arafat A.M. (2020). Comparative experimental investigation of natural fibers reinforced light weight concrete as thermally efficient building materials. J. Build. Eng..

[61] Hassan T., Jamshaid H., Mishra R., Khan M.Q., Petru M., Novak J., Choteborsky R., Hromasova M. (2020). Acoustic, Mechanical and Thermal Properties of Green Composites Reinforced with Natural Fibers Waste. Polymers.

[62] Mishra R., Gupta N., Pachauri R., Behera B.K. (2015). Modelling and simulation of earthquake resistant 3D woven textile structural concrete composites. Compos. Part B Eng..

[63] Mishra R. (2018). FEM based prediction of 3D woven fabric reinforced concrete under mechanical load. J. Build. Eng..

[64] Anggono J., Farkas A., Bartos A. (2019). Deformation and failure of sugarcane bagasse reinforced PP. Eur. Polym. J..

[65] Gu M., Ahmad W., Alaboud T.M., Zia A., Akmal U., Awad Y.A., Alabduljabbar H. (2022). Scientometric Analysis and Research Mapping Knowledge of Coconut Fibers in Concrete. Materials.

[66] Bunsell A.R. (2018). Handbook of Properties of Textile and Technical Fibres.

[67] Omoniyi T.E., Olorunnisola A.O. (2022). Effects of manufacturing techniques on the physico-mechanical properties of cement-bonded bagasse fiber composite. J. Nat. Fibers.

[68] Joseph L., Madhavan M.K., Jayanarayanan K., Pegoretti A. (2022). High Temperature Performance of Concrete Confinement by MWCNT Modified Epoxy Based Fiber Reinforced Composites. Materials.

[69] Xu J., Ma J., Zhang Q., Sugahara T., Yang Y., Hamada H. (2016). Crashworthiness of carbon fiber hybrid composite tubes molded by filament winding. Compos. Struct..

[70] Ma Y., Sugahara T., Yang Y., Hamada H. (2015). A study on the energy absorption properties of carbon/aramid fiber filament winding composite tube. Compos. Struct..

[71] Supian A.B.M., Sapuan S.M., Zuhri M.Y.M., Zainudin E.S., Ya H.H., Hisham H.N. (2021). Effect of winding orientation on energy absorption and failure modes of filament wound kenaf/glass fibre reinforced epoxy hybrid composite tubes under intermediate-velocity impact (IVI) load. J. Mater. Res. Technol..

[72] Chang Y., Zhou Y., Wang N., Lu K., Wen W., Xu Y. (2023). Micro-mechanical damage simulation of filament-wound composite with various winding angle under multi-axial loading. Compos. Struct..

[73] Pellegrin M.Z.D., Acordi J., Montedo O.R.K. (2021). Influence of the length and the content of cellulose fibers obtained from sugarcane bagasse on the mechanical properties of fiber-reinforced mortar composites. J. Nat. Fibers.

[74] Smith N., Virgo G., Buchanan V. (2008). Potential of Jamaican banana, coconut coir and bagasse fibres as composite materials. Mater. Charact..

